# Cervical Cancer Screening with DNA-HPV Testing and Precancerous Lesions Detection: A Brazilian Population-based Demonstration Study

**DOI:** 10.1055/s-0043-1763493

**Published:** 2023-03-06

**Authors:** Julio Cesar Teixeira, Diama Bhadra Vale, Michelle Garcia Discacciati, Cirbia Silva Campos, Joana Froes Bragança, Luiz Carlos Zeferino

**Affiliations:** 1 Universidade Estadual de Campinas, Faculty of Medical Sciences, Department of Obstetrics and Gynecology, Campinas, SP, Brazil

**Keywords:** cervical cancer, papillomavirus infections, cancer screening, pap smear, DNA-HPV test, câncer do colo do útero, infecções por papilomavírus, detecção precoce de câncer, teste de papanicolaou, teste de DNA para HPV

## Abstract

**Objective** To evaluate the rates of precancerous lesions, colposcopy referral, and positive predictive value (PPV) by age groups of a population-based screening with DNA-HPV testing.

**Methods** The present demonstration study compared 16,384 HPV tests performed in the first 30 months of the program with 19,992 women tested in the cytology screening. The colposcopy referral rate and PPV for CIN2+ and CIN3+ by age group and screening program were compared. The statistical analysis used the chi-squared test and odds ratio (OR) with 95% confidence interval (95%CI).

**Results** The HPV tests were 3.26% positive for HPV16-HPV18 and 9.92% positive for 12 other HPVs with a 3.7 times higher colposcopy referral rate than the cytology program, which had 1.68% abnormalities. Human Papillomavirus testing detected 103 CIN2, 89 CIN3, and one AIS, compared with 24 CIN2 and 54 CIN3 detected by cytology ( *p* < 0.0001). The age group between 25 and 29 years old screened by HPV testing had 2.4 to 3.0 times more positivity, 13.0% colposcopy referral, twice more than women aged 30 to 39 years old (7.7%; *p* < 0.0001), and detected 20 CIN3 and 3 early-stage cancer versus 9 CIN3 and no cancer by cytology screening (CIN3 OR= 2.10; 95%CI: 0.91–5.25; *p* = 0.043). The PPV of colposcopy for CIN2+ ranged from 29.5 to 41.0% in the HPV testing program.

**Conclusion** There was a significant increase in detections of cervix precancerous lesions in a short period of screening with HPV testing. In women < 30 years old, the HPV testing exhibited more positivity, high colposcopy referral rate, similar colposcopy PPV to older women, and more detection of HSIL and early-stage cervical cancer.

## Resumo

**Objetivo** Avaliar as taxas de lesões pré-cancerosas, encaminhamento para colposcopia e valor preditivo positivo (VPP) por faixas etárias de rastreamento populacional com teste DNA-HPV.

**Métodos** O presente estudo de demonstração comparou 16.384 testes de HPV realizados nos primeiros 30 meses do programa com 19.992 mulheres testadas no rastreio citológico. Os programas foram comparados por taxa de encaminhamento de colposcopia e VPP para NIC2+ e NIC3+ por faixa etária. A análise estatística utilizou o teste de qui-quadrado e odds ratio (OR, na sigla em inglês) com intervalo de confiança (IC) de 95%.

**Resultados** Os testes de HPV foram 3,26% positivos para HPV16-HPV18 e 9,92% positivos para 12 outros HPVs, com uma taxa de encaminhamento de colposcopia 3,7 vezes maior do que o programa de citologia, que teve 1,68% de anormalidades. O teste de HPV detectou 103 NIC2, 89 NIC3 e um AIS, em comparação com 24 NIC2 e 54 NIC3 detectados por citologia ( *p* < 0,0001). O rastreio por teste de HPV no grupo etário 25 a 29 anos teve 2,4 a 3,0 vezes mais positividade, 13,0% de encaminhamento para colposcopia, 2 vezes mais que mulheres de 30 a 39 anos (7,7%; *p* < 0,0001), e detectou 20 NIC3 e 3 cânceres em estágio inicial *versus* nove NIC3 e nenhum câncer pelo rastreio citológico (NIC3 OR= 2,10; 96%CI: 0,91–5,25; *p* = 0,043). O VPP da colposcopia para NIC2+ variou de 29,5 a 41,0% no programa de teste de HPV.

**Conclusão** Houve um aumento significativo na detecção de lesões pré-cancerosas do colo do útero em um curto período de rastreamento com teste de HPV. Em mulheres < 30 anos, o teste de HPV exibiu mais positividade, alta taxa de encaminhamento para colposcopia com VPP semelhante a mulheres mais velhas, e mais detecção de HSIL e de câncer cervical em estágio inicial.

## Introduction

In Brazil, 16,590 new cases of cervical cancer are expected annually and a 6.17 per 100,000 women mortality rate, which means that 1 woman dies of cervical cancer every 90 minutes. 1 A national screening program started in the 1980s and expanded significantly after that. The current Brazilian Guidelines for Cervical Cancer Screening (2016) recommends conventional cytology (Pap test) every 3 years in women aged 25 to 64 years old. 2 The Brazilian Unified Public Health System (SUS, in the Portuguese acronym) registers a yearly number of cytology tests performed sufficient to cover 80% of all targeted women. However, the estimated coverage does not exceed 30%, with a significant rate of excess tests as they are performed in women outside the age range and at an inadequate periodicity. 3 The mortality rates have not reduced, and > 60% of cervical cancer cases are diagnosed in advanced stages. 4 5

The World Health Organization (WHO), supported by the consolidated evidence since 2013, recommends that the new programs replace the cytology-based strategy with the Human Papillomavirus (HPV) test-based screening, starting at 30 years old. 6 Several studies and scientific associations indicate the use of HPV tests with caution in the age group up to 29 years old, due to possible limitations in achieving the expected efficacy. However, the age group between 25 and 29 years old is a frequent target of cervical cancer screening programs. 7 8 9 Our research group, seeking to produce evidence to support improving the current scenario, started a population-based demonstration study in 2017. The objective was to raise epidemiological, cost-benefit, and life gain indicators to support the transition of the current Brazilian cytology-based opportunistic screening to a DNA-HPV test-based organized screening program starting at 25 years old.

The “PREVENTIVO” program (PREvention of HPV Viruses in ENTire Indaiatuba by Vaccination and Organization of the screening) is based on primary DNA-HPV testing screening. It was implemented in Indaiatuba, a medium-sized city (250,000 inhabitants) in São Paulo State, Brazil. 10 Indaiatuba is included in the macro-region of Campinas, attended by the University of Campinas. It has health care facilities networked with information from computerized systems and individual digital records. A cost-effectiveness analysis based on real-life costs was published in 2021, showing that HPV testing every 5 years had lower costs than cytology every 3 years. 11 In sequence, just after 50% completion of the 1 ^st^ round in high coverage (> 80%), there was an impressive detection of 21 prevalent cancers, two-thirds as microinvasive carcinomas, a washing effect anticipating the diagnosis in 10 years. 12

The current paper presents the detection of precancerous lesions, particularly in women < 30 years old, during the first round of the ongoing population-based screening program with DNA-HPV testing, just before the pandemic started.

## Methods

The present study considered information from the first 30 months of the population-based demonstration study, an HPV test-based screening program, from October 2017 to March 2020, just before the impact of the pandemic. The aim was to verify the detection rate of cervical intraepithelial neoplasia grade 2 or 3 and worse (CIN2 + , CIN3 + ), the colposcopy referral rate, and the positive predictive value (PPV) of a colposcopy by age groups, comparing with the previous information from the cytology screening.

The HPV test-based screening protocol from the PREVENTIVO program was previously published. 10 Summarily, the program retrieves women between 25 and 64 years old, relying on the municipal public health system (50% of the total population). 13 Therefore, considering the population estimated of 70,573 women for 2020, the target population was 35,000 women. 14 There is no information about HPV vaccination in the screened women. However, the Brazilian National Vaccination Program started in 2014 for girls aged up to 13 years old, so women will reach the minimum age of 25 for screening in 2025. Therefore, we consider that close to 100% of the women studied were unvaccinated.

## HPV Test and Flowchart of the Screening Program

Based on the previously published validation study, the Cobas HPV Test (Roche Molecular Systems, Pleasanton, CA, USA) was adopted as the screening test. 15 This test simultaneously provides individual results on the highest risk genotypes – HPV 16 and HPV 18–and aggregate result (positive or negative) on the twelve other (12OT) high-risk (hr) HPV genotypes (types 31, 33, 35, 39, 45, 51, 52, 56, 58, 59, 66 and 68). All genotypes were tested simultaneously. Screening samples were collected at primary healthcare facilities, and the tests were performed at a regional laboratory. Clinical procedures according to the first result of the DNA-HPV test were:

1. Negative DNA-HPV test: advised to return after 5 years to repeat the test;
2. Positive HPV16 and/or HPV18 test: referred for colposcopy and biopsy if needed;
3. Positive DNA-HPV test for the 12OT hr-HPV: liquid-based cytology (LBC) was performed on the same sample. If a cytological abnormality (Atypical Squamous Cell of Undetermined Significance [ASC–US] or worse) was detected, the woman was referred for colposcopy. When the cytology was negative, the woman was advised to return after 12 months to repeat the DNA-HPV test.

The flagged women were referred to a colposcopy clinic. Excision of the transformation zone (ETZ) under local anesthesia was performed when needed. Histopathologic results were negative, low-grade (LSIL or CIN1) and high-grade intraepithelial lesions (HSIL or CIN2, CIN3, and adenocarcinoma in situ [AIS]), and cancer. Cases suspicious or diagnosed with cervical cancer or in a more complex clinical situation were referred to the regional gynecological cancer center at the Women's Hospital of the Universidade de Campinas, where oncological surgeries, radiotherapy, and chemotherapy are available.

The municipal health secretary managed the screening program at the central level. The PREVENTIVO program used algorithms according to study protocols, which did not allow a sample collection of a woman outside the established flowchart, avoiding testing twice the same woman or out of the recommendation. The research team from the Universidade de Campinas had access to all data and worked as a quality control surveillance system of the progress of the program. The present study obtained information about age, colposcopy, and histology results from all women with abnormal screening tests. The researchers do not interfere with the management or care of the screening program. Therefore, the level of organization of the PREVENTIVO program helps to achieve complete information about the procedures and results of women with a positive screening test. Fourteen women moved out of the city and were discharged by the program, following the regulatory recommendations and with adequate referral to the new city.

## Comparison Group: Cytology-based Screening

As a historical reference, information was obtained from the previous screening program with conventional cytology in Indaiatuba, performed in the period from October 2014 to March 2017, 30 months just before the new program started. The cytology results and information of the women were obtained from the regional Cytology Laboratory at the Universidade de Campinas, where 100% of tests from the SUS were read. The cytologic diagnoses followed the Bethesda Reporting System, 16 and some follow-up information are missing due to the lack of central regulatory coordination. Only one cytology test was considered per woman, the one with the worst diagnosis. The cytology program lacks information about the number of negative colposcopies and the total of colposcopies performed; the only available information was about positive colposcopies with histologic results from biopsy or ETZ performed (335 colposcopy referrals with 112 known diagnoses).

After the end of all regular procedures, the definitive diagnosis was considered the worst grade of the histological evaluation of tissue obtained from colposcopy-directed cervical biopsy or ETZ (CIN2+ and CIN3 + ). All CIN2 diagnoses in the PREVENTIVO program had an immunohistochemical confirmation with automatized biomarker p16INK4a detection (CINtec Histology; Ventana Medical Systems Inc, Tucson, AZ, USA).

The colposcopy referral rate and the CIN2+ and CIN3+ detection rates by age group were calculated in both screening programs with an odds ratio (OR) with a 95% confidence interval (CI). For the DNA-HPV test screening program, the number of colposcopies performed as well as the positive predictive value (PPV) of the colposcopy to detect CIN2+ or CIN3+ were calculated. The CIN2–3 detection rate by the positive screening test in the age group between 25 and 29 years old was calculated and compared with the age group between 30 and 64 years. The statistical analysis was performed with the chi-squared test using StatsDirect statistical software 3.0 (England, www.statsdirect.com ). P-values < 0.05 were considered significant.

The Ethics Committee of the University of Campinas approved the present study (number 1045580, May 1, 2015; CAAE: 43815315.9.0000.5404). The mayor of Indaiatuba sanctioned a law instituting the HPV test as the standard for screening in 2017, which replaced conventional cytology in public healthcare. 17

## Results

A total of 16,384 DNA-HPV tests in the PREVENTIVO program and 19,992 conventional cytology tests in the previous cytology screening were performed, considering 30 months between each program. The test outcomes by screening program are shown in Table 1 . There were 86.84% negative DNA-HPV tests, 3.26% positive by HPV16 and/or HPV18, and 9.92% positive for 12OT hr-HPV that performed LBC at the same sample. The cytology program exhibited 1.68% abnormal tests and 0.26% suggesting HSIL (CIN2–3). The colposcopy referral rate was 3.7 times higher in the DNA-HPV test program: 6.26 (1,026/16,384) versus 1.68 (335/19,992) in the cytology program ( *p* < 0.0001). The PREVENTIVO program had a compliance of 78% (788/1,012) of colposcopies performed, considering data until May 2021, while this information is unknown regarding the previous cytology program. The detection of CIN2+ was 1.31% in the PREVENTIVO program and 0.45% in the cytology program (rate = 2.9). The number of women with any lesion was 405 CIN1+ detected by DNA-HPV testing screening and 106 CIN1+ cases in the cytology program, which means 14 times more LSIL ( *p* < 0.0001) and 2 to 5 times more HSIL ( *p* < 0.0001) ( Table 1 ).

**Table 1:** Tests results according to the screening program

Outcome	Screening program						
	DNA–HPV test ( *n* = 16,384)		Outcome	Cytology ( *n* = 19,992)		*Rate*	*p–value* *
	n	*(%)*		n	*(%)*		
Test results			Test results				
Negative	14,228	86.84	Negative	19,657	98.32	–	< 0.0001
HPV16+ α	381	2.33	ASC-US #	224	1.12	–	
HPV18+ α	127	0.78	LSIL #	58	0.29	–	
HPV16+ and 18+ α	24	0.15	HSIL (CIN2)	28	0.14	–	
12OT hr-HPV+ and cytology positive	494	3.02	HSIL (CIN3)	24	0.12	–	
12OT hr-HPV+ and cytology negative	1,130	6.90	AGC	1	<0.01	–	
Colposcopy referral	1,026	6.26	Colposcopy referral	335	1.68	3.7	< 0.0001
Diagnosis completed £	788	4.81	Diagnosis completed α	112	0.56	–	
Negative	383	2.34	Negative β	6	0.03	–	
CIN1	191	1.17	CIN1	16	0.08	14.6	< 0.0001
CIN2	103	0.63	CIN2	24	0.12	5.3	< 0.0001
CIN3	90	0.55	CIN3	54	0.27	2.0	< 0.0001
Cancer §	21	0.13	Cancer	12	0.06	2.2	0.032

Abbreviations: 12OT hr-HPV, 12 other high-risk human papillomaviruses (types 31, 33, 35, 39, 45, 51, 52, 56, 58, 59, 66, and 68); AGC, atypical glandular cells; ASC-US, atypical squamous cell of undetermined significance; CIN, cervical intraepithelial neoplasia; LSIL-HSIL, low-grade or high-grade squamous intraepithelial lesions.

α HPV16 and/or 18 positive ± 12OT hr-HPV positive.

* Chi-squared test.

# Colposcopy indicated if two consecutive abnormal cytology tests in six-12 months intervals for ASC-US and in six months for LSIL. 2

£ Diagnosis completed: worse histologic result by biopsy or excision of the transformation zone.

β Negative in cytology program: six cases with biopsy negative, 215 cases with missing information (if colposcopy negative without biopsy or colposcopy not done), and eight cases 'unknown' (four dropped out and four with missed biopsy result).

§ Cancer cases were previously presented and discussed in detail. 12 DNA-HPV test program considered colposcopies performed until May 2021. It was added one Adenocarcinoma in situ (AIS) case into the CIN3 diagnosis.

Excluding 21 cervical cancers detected in the PREVENTIVO program and 12 cancer cases detected in the cytology program, the DNA-HPV screening detected more precursor lesions, 103 CIN2, 89 CIN3, and one AIS, compared with 24 CIN2 and 54 CIN3 detected in the cytology screening program ( *p* < 0.0001). The HPV16 colposcopy referral rate was 2.48, 39.5% (405/1,026) of all colposcopies indicated, and was 48% higher than the rate of colposcopy referrals for the entire cytology program. The 12OT hr-HPV positive test plus abnormal cytology had a colposcopy referral rate of 3.02, corresponding to 48.1% (494/1,026) of all indicated colposcopies.

Table 2 compares the programs regarding the colposcopy referral rate by age group. The highest colposcopy referral rates were associated with younger women in both programs. The age group between 25 and 29 years old had a 13.0% colposcopy referral rate, twice more than that of women aged between 30 and 39 years old (7.7%; *p* < 0.0001). In a similar analysis for the cytology program, the age group between 25 and 29 years old had a 3.0% colposcopy referral rate, almost twice more than that of women between 30 and 39 years old (1.7%; *p* < 0.001). Comparing the programs, the DNA-HPV test screening referred more colposcopies in all age groups, with similar ORs for the age groups between 25 and 29 years old (OR 4.80; 95%CI: 3.59–6.51) and between 30 and 39 years old (OR 4.81; 95%CI: 3.70–6.32). The cytology screening program tested more women outside the target age group: 16.2% ( *n* = 3,249) up to 24 years old with a 3.0 to 3.7% of colposcopy referral rate and eight CIN3 detected (no cancer); and 4.8% ( *n* = 995) of women ≥ 65 years old, with no abnormalities detected ( Tables 2 and 3 ).

**Table 2:** Distribution of women screened and colposcopy referral by age group and screening program

Age group (years old)	Screening program								Colposcopy referral OR (95%CI)	
	DNA–HPV test				Cytology					
	Women screened		Colposcopy referral		Women screened		Colposcopy referral #			
	*n*	(%)	*n*	(%)	*n*	(%)	*n*	(%)		
≤ 19	0	(0)	–	–	1,227	(6.1)	46	(3.7)	–	
20–24	54	(0.3)	11	(20.4)	2,022	(10.1)	60	(3.0)	8.37	[3.69–17.5]
25–29	2,117	(12.9)	275	(13.0)	1,992	(10.0)	60	(3.0)	4.80	[3.59–6.51]
30–39	4,351	(26.6)	335	(7.7)	4,162	(20.8)	71	(1.7)	4.81	[3.70–6.32]
40–49	4,478	(27.3)	233	(5.2)	4,275	(21.4)	64	(1.5)	3.61	[2.72–4.86]
50–64	5,267	(32.1)	165	(3.1)	5,349	(26.8)	34	(0.6)	5.06	[3.47–7.56]
≥ 65	117	(0.7)	7	(6.0)	965	(4.8)	0	(0.0)	–	
Total	16,384	(100.0)	1,026	(6.26)	19,992	(100.0)	335	(1.68)	3.92	[3.46–4.46]

# Colposcopy indicated if two consecutive abnormal cytology tests with ASC-US in six-12 months intervals or LSIL in six months. (2) OR (95%CI): odds ratios (95% confidence interval) for colposcopy referral by DNA-HPV test, with *p* < 0.0001 for all age groups. Colposcopy referral rate for 25–29 years versus 30–39 years in both programs had a Chi-squared test with *p* < 0.001. Excluded two clinical and/or symptomatic cervical cancer cases detected in women aged +65 years in the cytological screening period.

**Table 3:** Distribution of the final diagnoses according to the screening program and age group

Age group (years old)	Final diagnoses by the screening program									
	DNA–HPV test ( *n* = 788)					Cytology test ( *n* = 112)				
	Neg	CIN1	CIN2	CIN3	Cancer	Neg	CIN1	CIN2	CIN3	Cancer
≤19	*-*	–	*-*	*-*	–	0	1	6	1	0
20–24	3	2	1	1	0	1	4	1	7	0
25–29	106	51	32	20	3	1	5	7	9	0
30–39	104	63	38	38 *	6	0	4	4	23	5
40–49	81	45	23	20	9	2	1	4	6	2
50–64	84	28	9	11	3	2	0	2	8	3
≥ 65	5	2	0	0	0	0	0	0	0	2
Total	383	191	103	90 *	21	6	16	24	54	12

Abbreviations: CIN, cervical intraepithelial neoplasia; Neg, negative.

* Added one case of Adenocarcinoma in situ (AIS). Diagnoses: the worse histologic result by biopsy or excision of the transformation zone. DNA-HPV test program considered all colposcopies performed until May 2021. Cytology program: information available only for cases with the histologic result (missing information about negative colposcopy, without biopsy). Odds ratio for CIN3 in HPV testing screening: age group of 25–29y = 2.10 [0.91–5.25], *p* = 0.043; age group of 30–39 years = 1.59 [0.92–2.79], *p* = 0.052.

The cervical lesions detected by age group in each program are shown in Table 3 . The PREVENTIVO program screened 2,117 women aged between 25 and 29 years old with HPV test, detected 32 CIN2, 20 CIN3, and 3 cervical cancer cases: (1) microinvasive squamous-cell carcinoma, (1) microinvasive adenocarcinoma, (1) invasive adenocarcinoma Stage IB1. Comparatively, the results of 1,992 women screened in the cytology program in the same age group (25 to 29 years old) showed 7 CIN2, 9 CIN3, and no cancer (CIN3 OR= 2.10; 95%CI: 0.91–5.25; *p* = 0.043). The age group between 30 and 39 years old had the highest performance of the tests to detect CIN3 in both programs: 38/4,351 (0.87%) women screened by HPV test and 23/4,162 (0.55%) women screened by cytology (OR= 1.59; 95%CI: 0.92–2.79; *p* = 0.052). In the age group ≥ 65 years old, no CIN2–3 was detected in both programs, even including lesions detected in the 965 women screened by the cytology program. The colposcopy performance regarding the HPV test positivity exhibited the detection of 58 CIN3 (PPV 19.3%) cases with HPV16 and/or 18 positives (without cytology) and 32 CIN3 (PPV 12.5%) with 12OT hr-HPV positive with abnormal cytology ( *p* = 0.029). The CIN2–3 detection rate in women with an HPV positive test was 24 to 26%, with a similar performance comparing HPV16, HPV18, and 12OT hr-HPV plus abnormal cytology ( Table 4 ). Considering the CIN3 cases, the detection rate for HPV16 was 14.3%, similar to 12.1% for HPV 18 ( *p* = 0.544) and higher than the 9.2% for the 12OT hr-HPV plus abnormal cytology ( *p* = 0.04). Human Papillomavirus 6 was associated with 50% (46/92) of the CIN3 cases, 3.4 times more than HPV18 (15.2%; 14/92) and 1.4 times more than 12OT hr-HPV plus abnormal cytology (34.8%; 32/92).

**Table 4:** Detection of CIN according to the HPV screening test

Diagnosis	Positive screening test							
	HPV16 ( *n* = 321)		HPV18 ( *n* = 116)		12OT + LBC ( *n* = 347)		Total ( *n* = 784 * )	
	*n*	%	*n*	%	*n*	%	*n*	%
Negative	163	50.8	59	50.9	169	48.7	391	49.9
CIN1	74	23.1	28	24.1	94	27.1	196	25.0
CIN2	38	11.8	15	12.9	52	15.0	105	13.4
CIN3	46	14.3	14	12.1	32	9.2	92	11.7
CIN2 + CIN3	84	26.2	29	25.0	84	24.2	197	25.1

* Number of cases counted twice for HPV16 and 18 positive tests: 8 Negative, 5 CIN1, 2 CIN2, 2 CIN3, and 17 in the 'Total'. In the CIN3 diagnosis was considered 1 AIS by HPV16. The 21 cancer cases were not considered. Chi-square test: CIN2 + CIN3 for HPV16 versus 12OT ( *p* = 0.560) and for HPV18 ( *p* = 0.806); CIN3 for HPV16 versus 12OT ( *p* = 0.04) and for HPV18 ( *p* = 0.544). Note: HPV16 or 18: positive tests regardless 12OT (12 other high-risk HPV) status. CIN1–3, cervical intraepithelial neoplasia grade 1, 2, or 3; hr-HPV, high-risk human papillomavirus; LBC (liquid-based cytology) positive if ASC-US or worse.

The colposcopies performed in the *PREVENTIVO* program had a similar positive screening test distribution by age group ( Table 5 ). In the targeted age range for the screening (25 to 64 years old), the PPV for CIN2+ ranged from 29.5 to 41.0%, and the PPV for CIN3+ ranged from 14.3 to 22.5%. The highest PPVs were in the age groups between 30 and 39 and between 40 and 49 years old. According to the final diagnosis, HPV 16 was more prevalent (41.3%; 333/806) with a higher association with CIN2+ diagnosis (28.8%; 96/333). However, the 12OT hr-HPV positive tests with LSIL/ASC-US cytology showed 19.2% (85/443) of CIN2–3 and 4 cervical cancers cases (data not shown). Few colposcopies were performed outside the targeted age range with detection 1 CIN2 and 1 CIN 3, among 7 colposcopies performed in women ≤ 24 years old. No HSIL was detected in 7 colposcopies performed in women ≥ 65 years old.

**Table 5:** Final diagnosis by HPV screening test, age group, and positive predictive value (PPV) of colposcopy to detect precancerous lesion

Age group (years old)	Final diagnosis by screening test ( *n* = 788)							PPV of colposcopy			
	HPV16 # positive		HPV18 # positive		12OT hr-HPV and LBC positives		Total	CIN2+		CIN3+	
	*n*	(%)	*n*	(%)	*n*	(%)	*n*	*n*	PPV	*n*	PPV
20–24	4	(1.2)	1	(0.8)	2	(0.6)	7	2	40.0	1	20.0
25–29	96	(28.8)	32	(26.2)	91	(25.8)	219	55	34.2	23	14.3
30–39	98	(29.4)	40	(32.8)	116	(32.9)	254	82	41.0	44	22.0
40–49	73	(21.9)	26	(21.3)	83	(23.5)	182	52	40.3	29	22.5
50–64	59	(17.7)	22	(18.0)	58	(16.4)	139	23	29.5	14	17.9
≥ 65	3	(0.9)	1	(0.8)	3	(0.8)	7 ^α^	0	–	0	–
Total	333	(100.0)	122	(100.0)	353	(100.0)	808 *	214	37.1	111	19.2

# HPV16 or 18: positive tests regardless 12OT (other) hr-HPV status.

* 20 cases tested positive for both, HPV 16 and 18 (counted twice). Note: colposcopy done until May 2021; 14 dropped out and 224 waiting. LBC (liquid-based cytology) positive if ASC-US or worse; CIN2+/3+, cervical intraepithelial neoplasia grade 2 or 3 and worse (including cancer cases); hr-HPV, high-risk human papillomavirus.

The age group between 25 and 29 years old had 2.4 to 3.0 times more HPV testing positive than women between 30 and 64 years old, with an OR of 2.1 to 3.0 for CIN2–3 lesions diagnosed according to the HPV type detected ( Table 6 ). There was no difference in the proportion of CIN2–3 detected by HPV type and age group among positive tests, ranging from 23.3 to 27.1%.

**Table 6:** Detection of CIN2–3 in women aged 25 to 29 years old by positive HPV screening test compared with older age groups

Outcome	Age group				OR (95%CI)	*p-value*
	25–29 years old		30–64 years old			
	*n*	%	*n*	%		
Women screened	2,114	12.92	14,078	86.04		
Positive test						
HPV16 ( *n* = 314)	96	4.54	218	1.55	3.02 (2.34–3.88)	< 0.0001
CIN2–3 ( *n* = 83)	26	1.23	57	0.41	3.06 (1.84–4.96)	< 0.0001
CIN2–3 per test+	26/96	27.1	57/218	26.2		
HPV18 ( *n* = 114)	30	1.42	84	0.60	2.40 (1.52–3.69)	0.0001
CIN2–3 ( *n* = 29)	7	0.33	22	0.16	2.12 (0.76–5.15)	0.075
CIN2–3 per test+	7/30	23.3	22/84	26.2		
12OT HPV + LBC ( *n* = 342)	90	4.26	252	1.79	2.47 (1.91–3.17)	< 0.0001
CIN2–3 ( *n* = 83)	21	0.99	62	0.44	2.27 (1.31–3.78)	0.002
CIN2–3 per test+	21/90	23.3	62/252	24.6		

Abbreviations: CIN2–3, cervical intraepithelial neoplasia grade 2 or 3; hr-HPV, high-risk human papillomavirus; LBC, liquid-based cytology, positive if ASC-US or worse; OR (95%CI), odds ratios (95% confidence interval).

Note: excluded 21 cervical cancer cases; HPV16 or 18: positive tests regardless 12OT (12 other high-risk HPV) status, and 17 cases tested positive for both, HPV16 and 18. Diagnosis completed by age group: 79.6% (219/275) for 25–29y and 78.4% (575/733) for 30–64y.

## Discussion

The present article describes the first 30 months of a screening program with primary HPV testing, organized and implemented into routine practice, outside the research setting, compared with a previous program based on conventional cytology. The HPV-based program performed fewer screening tests than the cytology screening with high age compliance, detecting 2.9 times more CIN2 + , which means 193 CIN2–3 lesions compared with 78 cases detected in the previous cytology screening. These achievements in unvaccinated women were associated with a 3.7 times higher colposcopy referral rate for the new program. In summary, there was 13.2% (2,156/16,384) of positive HPV testing, with 6.26% (1,026/16,384) of colposcopy referral, and 1.31% of CIN2+ detected.

The detection of CIN2–3 is the primary goal of screening programs since 20 to 50% of them have the potential to progress to invasive lesions. 18 19 20 This more significant number of HSIL detected is associated with the higher sensitivity of the HPV-DNA test, 9 15 and the expansion of the population coverage, with a 90% projection of the SUS target population, as previously published. 12 The compliance with the flowchart of the program was high, with 78% of all colposcopies indicated already performed, which can be considered a milestone of the program. In a similar implementation screening study in Argentina (2011), with 49,000 HPV tests performed between 2012 and 2014, the researchers described a 74.6% of colposcopy compliance rate. 21

It is important to note that most women screened with the HPV test had been participating in the previous screening with cytology, which is standard in the country. Although the numbers of lesions detected were high, a significant reduction may be expected in the next round of the program, including the colposcopy referral rate, as already demonstrated in other countries with ongoing programs. 22 23 24

From the higher colposcopy referral rate observed in the demonstration program, 51.9% (532/1026) were indicated by HPV16 and/or 18 positive tests. According to the program guideline, in these cases, the colposcopy is performed without any cytology information, which can be considered an additional challenge for the colposcopists. Nevertheless, the colposcopies performed in the PREVENTIVO program showed higher CIN3 PPV for HPV16 and/or 18 positive tests (19.3%) compared with the cases from the 12OT hr-HPV positive with abnormal cytology (12.5%). There were five times more CIN2, and twice more CIN3 detected in the HPV testing compared with the cytology-based screening.

Although the cytological result can help the colposcopic evaluation and even partially reduce the colposcopy referral, 25 26 colposcopy for HPV16/18 positive test was performed without cytology. This decision is based on the higher risk for HSIL- or cancer-related, the ability to enable risk stratification for women aged 25 to 29 years old, 15 and the program flowchart simplification, avoiding issues related to coexisting two different tests, the conventional cytology and HPV test with LBC. The CIN2–3 detection rate in women with HPV positive tests was 24 to 26%, with a similar performance comparing the HPV16, HPV18, and the 12OT hr-HPV plus abnormal cytology. Human Papillomavirus 16 was associated with 50% of CIN3-AIS cases, 1.4 to 3.4 times more than other HPV types.

Another concern of the study was the screening performance of the HPV test in the age group between 25 and 29 years old, considered a novelty at the beginning of the PREVENTIVO program. This age group exhibited 22.3% positive screening tests (473/2,117) and a colposcopy referral rate of 13.0%. It was almost double the 7.7% for the age group between 30 and 39 years old, and a PPV for CIN2+ of 34.2%. Although the cytology program exhibited a similar double of colposcopy referred to younger women (3.0% versus 1.7% for between 30 and 39 years old), there was 5 times less. When comparing the programs, the OR was equivalent between the age groups between 25 and 29 and between 30 and 39 years old. Similarly, a recently published population-based UK study evaluating the screening performance with HPV testing in unvaccinated women reported 26.9% of positive tests and 10.4% of directly referred to colposcopy for the age group between 24 and 29 years old. 26 A recent review of the French program with primary HPV testing stated that lesions in young women such as CIN2 have a high proportion of regression and that they could dispense an immediate colposcopy and wait for a repeat HPV test in 12 months. 24 Nevertheless, these results are influenced by the population prevalence of HSIL and cancer and, shortly, by the proportion of women adequately vaccinated against HPV. Our study proposed a single flowchart for all age groups. Although there was a high colposcopy referral rate, the HPV test in the age group between 25 and 29 years old demonstrated a higher performance, detecting 52 CIN2–3 and 3 cervical cancers at early stages, compared with 16 precursor lesions and no cancer detected by the cytology screening.

The PREVENTIVO program exhibited a colposcopy PPV of 37.1% for CIN2+ and 19.2% for CIN3+ for all age groups, slightly lower than that reported for the British study with 43 and 27%, respectively, the last using cytology triage to colposcopy referral for all HPV positive tests. 26

The present study is the first population-based demonstration program to replace conventional cytology with a DNA-HPV test for primary screening in a real-life scenario in the Brazilian public health system. The program structure and its results add evidence to the previously published information about coverage, program compliance, early-stage cervical cancer detection, 12 and cost-effectiveness. 11 It supports the adoption of the HPV test for a new national screening program.

The limitation of the present study is the use of a historical cohort as a comparison. There was a significant lack of detailed information about the previous cytology-based screening program, such as information about the management of positive tests. However, comparing the colposcopy referral rate and the cases with histologic results available from cervix procedures was possible. Another limitation was that the PREVENTIVO program considered only the SUS users, which comprise 50% of the women population.

The strength of the present study was the organization of the PREVENTIVO screening program in implementation providing access to crucial information for surveillance, not available in the opportunistic programs. The new program applies processes such as a simplified flowchart, and a computerized central management system. It is being performed in the same health units, by the same staff, and in the same population as the previous program, which highlights the results achieved, indicating that they are related to the process of the new program.

The results presented are preliminary but the best possible without the influence of the pandemic that started in Brazil in March 2020. The effect of the pandemic on the program will be the subject of an ongoing detailed analysis.

All results reported from the present demonstration study are highly impactful, even with the short program running time. The question remains on how to expand this action to other regions of Brazil, where the current situation could be worse. As the PREVENTIVO program used all the existing structures for the cytological program and simplified the work process, we believe the program can be replicated in all urban areas of the country. The main challenge to its implementation is the initial budgetary impact of the cost of HPV testing, developing a system to integrate the health units, and creating a digital record with information from the population to manage and monitor the program. The first round of the PREVENTIVO program will finish in late 2022 and additional rounds will be necessary to confirm the findings. Keeping the program under monitoring can serve as a sentinel study for further regions starting their new program, assessing any additional actions needed, and establishing an effective and long-lasting program.

## Conclusion

There was a significant increase in detections of cervical cancer precursor lesions in a short period after the shift from opportunistic screening with conventional cytology to an organized program with HPV DNA test. In addition, women < 30 years old screened by HPV testing exhibited more positive tests and a higher colposcopy referral rate. However, the PPV of colposcopy was similar to that of older women, with more HSIL and early-stage cervical cancer cases detected.
